# Molecular Classification of Enteroviruses Not Identified by Neutralization Tests

**DOI:** 10.3201/eid0803.010200

**Published:** 2002-03

**Authors:** Hideyuki Kubo, Nobuhiro Iritani, Yoshiyuki Seto

**Affiliations:** Osaka City Institute of Public Health and Environmental Sciences, Osaka, Japan

**Keywords:** enteroviruses, phylogenetic analysis, VP4 nucleotide sequences

## Abstract

We isolated six viruses from patients diagnosed with aseptic meningitis or hand, foot, and mouth disease. The cytopathic effect of these viruses on cultured cells was like that of enteroviruses. However, viral neutralization tests against standard antisera were negative. Phylogenetic analysis with the complete VP4 nucleotide sequences of these 6 viruses and 29 serotypes of enteroviruses classified 3 of the viruses as serotype echovirus type 18 (EV18) and 3 as serotype human enterovirus 71 (HEV71). These results were confirmed by remicroneutralization tests with HEV-monospecific antisera or an additional phylogenetic analysis with the complete VP4 nucleotide sequences. Phylogenetic analysis with complete VP4 genes is more useful than neutralization tests with enterovirus serotype-specific antisera in identifying enterovirus serotypes.

The human enterovirus (HEV) genus of the family Picornaviridae includes the human pathogens that cause a wide spectrum of acute disease, including hand, foot, and mouth disease (*1*), aseptic meningitis (*2*,*3*), encephalitis (*3*–*6*), and neonatal sepsislike disease (*7*,*8*). Sixty-four serotypes of HEV have been recognized antigenically by neutralization tests with anti-HEV antibodies (*9*). HEVs have long been classified on the basis of serotype-specific antisera in virus neutralization tests (*1*,*10*), the only method available for serotyping HEVs. However, virus neutralization is both labor- and time-intensive, and antigenic variants in many serotypes of HEV can affect test results (*1*).

The HEV genome comprises a 5' nontranslated region (NTR), a long open reading frame that encodes a protein of approximately 2,100 amino acid residues, a short 3' NTR, and a polyadenylated tail. The polyprotein is co- and post-translationally cleaved to yield four structural proteins: VP4, VP2, VP3, and VP1 (*1*). Recently, attempts have been made to classify the HEV serotypes by using the partial nucleotide sequences of the HEV genomes (i.e., the 5' NTR [*11**-**13*], the VP4-VP2 junction [*14**-**16*], and VP1 [*17**-**20*]). Methods for molecular classification of HEVs should not only identify the serotypes rapidly but also detect antigenic variant strains or new serotypes. A new serotype of HEV has recently been identified by comparing the complete VP1 nucleotide sequences; its proposed name is HEV73 (*19*).

To investigate the HEV serotypes of six HEV-like viruses that were not neutralized by standard HEV typing sera, we determined the complete VP4 nucleotide sequences of these 6 viruses and 21 HEV antigenically defined serotypes, then performed phylogenetic analysis with another 8 HEV serotypes available from GenBank. The classifications of the untypeable viruses were confirmed by using HEV-monospecific antisera or an additional phylogenetic analysis with the VP4 sequences. The molecular classification of HEV with the complete VP4 sequences is useful for identifying the HEV serotypes.

## Methods

### Virus Isolation and the Neutralization Test

The clinical specimens were injected into Vero, RD-18S, or MA104 cells to isolate viruses. All cells were grown in minimum essential medium (MEM) containing 10% fetal bovine serum (FBS) and maintained in MEM containing 1% to 2% FBS after being added to 48-well plates (Sumitomo Bakelite, Tokyo, Japan). The cells were incubated for 1 week, after which culture fluids were passaged and incubated for another week. Cultured cells showing cytopathic effects were regarded as virus isolation-positive and, together with the culture supernatant, were harvested and stored at -80°C before use. To serotype the viruses, microneutralization tests were performed with antiserum pools of Lim and Benyesh-Melnick (*21*) (Denka Seiken, Tokyo, Japan) or in-house monospecific immune sera against coxsackie virus A10 (CAV10), CAV16, and HEV71, respectively.

### Viruses

Of the six viruses that could not be identified by the neutralization tests described above (Table 1), strains OC/0071, OC/0073, and OC/00272 were isolated from patients diagnosed with aseptic meningitis by using RD-18S cells. OC/00219, OC/00260, and OC/00261 were isolated from patients diagnosed with hand, foot, and mouth disease or aseptic meningitis by using Vero cells. No sera from these patients was available for analysis. Twenty-one serotypes were isolated and identified in our laboratory during 1995-2000 (Table 2); these strains were used in the experiments. For additional investigations of HEV71, we used eight HEV71 strains isolated and identified in our laboratory (Table 3).

**Table 1:** Unidentified enterovirus strains and patient information, Osaka, Japan, 2000

Strain	Patient age (years)	Specimen	Date of sampling	Clinical symptoms	Isolated cells
OC/0071	2	Stool	5/11/2000	AM^a^	RD-18S
OC/0073	2^b^	CSF	5/11/2000	AM	RD-18S
OC/00219	0	Throat swab	7/7/2000	HFMD	Vero
OC/00260	0	Throat swab	7/18/2000	HFMD, AM	Vero
OC/00261	0^c^	Stool	7/18/2000	HFMD, AM	Vero
OC/00272	6	Stool	7/18/2000	AM	RD-18S

^a^AM = aseptic meningitis; CSF = cerebrospinal fluid; HFMD = hand, foot, and mouth disease. ^b^Same patient as OC/0071. ^c^Same patient as OC/00260.

**Table 2:** Characteristics of 21 human enterovirus (HEV) serotypes antigenically defined, Osaka, Japan, 1995–2000

HEV serotype	Strain	Age (years)	Specimen	Date of sampling	Isolated cells
PV1	OC/00417	0	Throat swab	10/13/2000	Vero
PV2	OC/00138	0	Stool	6/10/2000	Vero
PV3	OC/99355	0	Nasal mucus	11/8/1999	Vero
EV3	OC/00467	7	Stool	11/13/2000	RD-18S
EV6	OC/99350	0	Stool	11/8/1999	RD-18S
EV7	OC/96221	7	Throat swab	7/22/1996	MA104
EV9	OC/00129	3	CSF	6/8/2000	RD-18S
EV11	OC/98535	3	Stool	9/23/1998	RD-18S
EV16	OC/95378	1	Throat swab	9/11/1995	MA104
EV18	OC/99-Hanasaka	7	Stool	11/8/1999	RD-18S
EV25	OC/00263	0	Stool	7/17/2000	RD-18S
EV30	OC/97633	1	Stool	9/29/1997	RD-18S
CAV9	OC/96234	4	CSF^a^	8/2/1996	RD-18S
CAV16	OC/00351	NA	Throat swab	8/31/2000	Vero
CBV1	OC/00364	0	CSF	9/6/2000	Vero
CBV2	OC/99284	0	Stool	9/11/1999	RD-18S
CBV3	OC/97620	6	CSF	9/19/1997	RD-18S
CBV4	OC/00362	1	Stool	9/8/2000	Vero
CBV5	OC/00223	0	Throat swab	7/7/2000	Vero
CBV6	OC/00325	0	CSF	8/8/2000	Vero
HEV71	OC/00168	2	Throat swab	6/21/2000	Vero

^a^ CSF = Cerebrospinal fluid; NA = not available.

**Table 3:** Characteristics of eight human enterovirus (HEV)71 strains, as antigenically defined, Osaka, Japan, 2000

Strain	Age (years)	Specimen	Date of sampling	Clinical symptoms	Isolated cells
OC/9632	NA^a^	Stool	4/11/1996	HFMD	MA104
OC/99-Ikeda	6	Stool	9/11/1999	HFMD, AM	Vero
OC/0078	1	Throat swab	5/18/2000	HFMD, AM	Vero
OC/0080	NA	Stool	5/9/2000	Diarrhea	Vero
OC/0096	5	CSF	5/25/2000	Diarrhea, AM	Vero
OC/00114	0	CSF	5/31/2000	Fever	Vero
OC/00125	6	Throat swab	6/7/2000	Encephalitis	Vero
OC/00168	4	Throat swab	6/21/2000	Herpangina	Vero

^a^ NA = Not available; HFMD = Hand, foot, and mouth disease; AM = aseptic meningitis; CSF = cerebrospinal fluid.

### RNA Extraction and Reverse Transcription

Viral RNAs were extracted from the cell-culture supernatants by using ISOGEN-LS (Nippon Gene, Tokyo, Japan). cDNAs were synthesized with an Omniscript Reverse Transcriptase Kit (QIAGEN K.K., Tokyo, Japan) according to the manufacturer’s instructions. The primers used for the synthesis were EVP-2 (5'-CCTCCGGCCCCTGAATGCGGCTAAT-3' relative to nt 444-468 in the genome of Poliovirus (PV) Sabin 1 strain) (*22*) and OL68-1 (5'-GGTAAYTTCCACCACCANCC-3' relative to nt 1178-1197 of Sabin 1), as described (*23*).

### Polymerase Chain Reaction Amplification of cDNAs

Polymerase chain reaction (PCR) was performed by using 2 μL of each cDNA in a 50-μL reaction mixture containing 1.5 U of Taq DNA polymerase (Takara Shuzo, Shiga, Japan), 20 pmol of EVP-2 primer, and 20 pmol of OL68-1 primer. Each reaction was incubated in a GeneAmp 9700 thermal cycler (Applied Biosystems, Foster City, CA) according to the following protocol: 5 minutes at 95°C, 40 cycles of 95°C for 30 seconds, 68°C for 30 seconds, 72°C for 1 minute, and then at 72°C for 5 minutes. After the appearance of approximately 750 bp-specific amplified fragments was confirmed by agarose gel electrophoresis, the amplicons were purified with a QIAquick PCR purification kit (QIAGEN).

### DNA Sequence Analysis

Approximately 100 ng of purified amplicon was used in the reaction with the BigDye Terminator Cycle Sequencing FS Ready Reaction Kit (Applied Biosystems), and DNA sequencing was performed by using an ABI PRISM 310 DNA sequencer (Applied Biosystems). All DNA sequencings were performed on both strands using EVP-4 (5'-CTACTTTGGGTGTCCGTGTT-3' relative to nt 541-560 in the genome of PV Sabin 1 strain) as the forward primer and OL68-1 as the reverse primer (*23*). Sequencer software (version 3.0; Hitachi Software, Tokyo, Japan) was used to determine the approximately 600-bp nucleotide sequence spanning 5' NTR to one third of VP2 (including all of VP4), translate nucleotide sequence to amino acid sequence, and decide the complete VP4 coding sequence of each virus.

### Phylogenetic Analysis

A phylogenetic tree based on the complete VP4 nucleotide sequence was constructed by the neighbor-joining method (*24*) as implemented with the CLUSTAL X program (version 1.63b, December 1997; http://www-igbmc.u-strasbg.fr/BioInfo/ClustalX/). The reliability of the neighbor-joining tree was estimated by bootstrap analysis with 1,000 pseudoreplicate datasets. The complete VP4 sequences of eight HEV serotypes not isolated in our laboratory were obtained from GenBank and included in the HEV analysis. Complete VP4 nucleotide sequences of another 18 HEV71 strains were obtained from GenBank and used in the phylogenetic analysis.

### Remicroneutralization Tests

According to results of HEV phylogenetic analysis with the complete VP4 nucleotide sequences, remicroneutralization tests using monospecific antiserum against echovirus 18 (EV18; Denka Seiken), or HEV71 (anti-HEV71/BrCr and anti-HEV71/C7 sera; both supplied by the National Institute of Infectious Diseases, Japan) were performed to confirm the serotype of the untypeable strains from the first microneutralization assay.

### Complete VP1 Nucleotide Compared with Deduced Amino Acid Sequences of HEV71 Strains

The complete VP1 nucleotide sequences of HEV71 strains OC/00168, 0C/00219, OC/00260, and OC/00261 were determined by the same procedure described above, except for the primers. The primers used for the analysis of VP1 nucleotide sequence were 71F2399 (5' -AGAAYTTYACCATGAAACTG-3' relative to nt 2380-2399 in the genome of HEV71 MS/7423/87 strain [25]; the nucleotide positions of the following are also relative to this strain: 71F2793 (5'-AGACATAACTGGYTACGCCAC-3' nt 2774-2793) and 71F3042 (5'-CATGTCACCYGCGAGCGCTT-3' nt 3023-3042) as the forward, 71R2712 (5'-CTACCAARCCTGCCCTACTG-3' nt 2693-2712), 71R3066 (5'-GGTACCCGTCGTAAAACCAC-3' nt 3047-3066) and 71R3376 (5'-AAGTTGCCCACGTAGATGGC-3' nt 3357-3376) as the reverse. The VP1 nucleotide sequence of HEV71 BrCr strain (*25*) was obtained from GenBank. Sequencer software (version 3.0; Hitachi Software) was used for determination and comparison of the complete VP1 nucleotide and deduced amino acid sequences of these HEV71 strains.

## Results

### Determination of Complete VP4 Nucleotide Sequences of HEVs

During May to July 2000, six viruses submitted to our laboratory (OC/0071, OC/0073, OC/00219, OC/00260, OC/00261, and OC/00272) could not be neutralized by standard pools of HEV typing sera and three antimonospecific sera (Table 1). However, the cytopathic effects of these viruses on RD-18S or Vero cells were all HEV-like (data not shown). To identify the serotypes of these untypeable HEV-like viruses by a method other than the neutralization assay, we determined the complete VP4 nucleotide sequences of all 6 strains and another 21 HEV serotypes identified in our laboratory over the past 6 years. The 3' end of the VP4 gene of each virus was determined from the deduced amino acid sequences as described (*26*,*27*). The complete VP4 nucleotide sequences of all HEV strains used in this study were 207 nt long, and the deduced amino acid sequences of all VP4 proteins were 69 amino acids long (data not shown).

### Phylogenetic Analysis of HEVs

**Figure 1:**
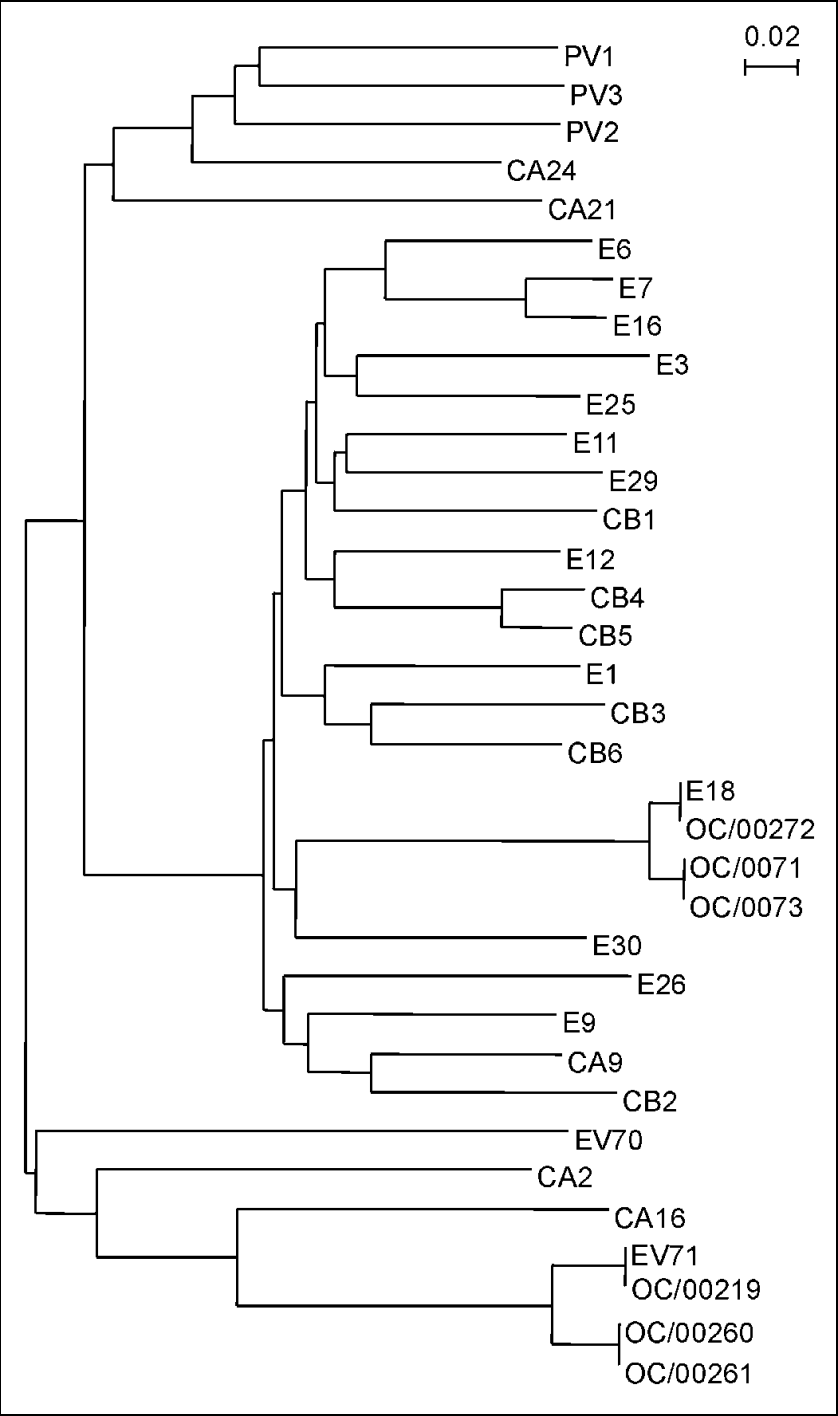
Phylogenetic analysis based on the human enterovirus (HEV) VP4 nucleotide sequences. The phylogenetic tree was constructed by the neighbor-joining method as implemented in CLUSTAL X program (version 1.63b). The marker denotes a measurement of the relative phylogenetic distance. The VP4 sequences of eight HEV serotypes described below are available from GenBank. The strain name and accession number are shown in parentheses: HEV1 (Bryson, AF250874), HEV12 (Travis, NC 001810), echovirus 26 (EV26, Coronel, AF117697), EV29 (JV-10, AF117698), coxsackie virus A2 (CAV2; Epsom/14448/99, AJ2296215), CAV21 (Coe, NC 001428), CAV24 (EH24/70, D90457), and HEV70 (J670/71, D00820).

A phylogenetic tree was constructed based on the complete VP4 nucleotide sequences of the 6 HEV-like untypeable strains, the 21 HEV serotypes identified in our laboratory as prototype strains, and another 8 HEV serotypes available from the GenBank database (Figure 1). The 29 different HEV serotypes defined antigenically were clustered in four distinct lineages, as described (*23*). Three of the six untypeable strains (OC/0071, OC/0073, and OC/00272) were classified nearest to EV18. The VP4 nucleotide sequences of strains OC/0071 and OC/0073 were identical. The VP4 gene sequence of OC/00272 was the same as that of OC/99-Hanasaka, which was used as a prototype strain for EV18. The nucleotide sequences of these two clusters differed by 5 nt, but the deduced amino acid sequences were the same (data not shown). The other three untypeable strains (OC/00219, OC/00260, and OC/00261) were classified nearest to HEV71. The VP4 sequence of OC/00219 was the same as that of OC/00168, which was used as a prototype strain for HEV71. The VP4 nucleotide sequences of OC/00260 and OC/00261 were identical. The difference between these two clusters was 11 nt. The deduced amino acid sequences were the same (data not shown).

### Remicroneutralization Tests

According to the results of the phylogenetic analysis based on the complete VP4 nucleotide sequences, remicroneutralization tests were performed. Microneutralization tests using the monospecific immune serum for EV18 were done against OC/0071, OC/0073, and OC/00272, and this serum neutralized these viruses. The same tests, using the two species of monospecific immune serum, anti-HEV71/BrCr and anti-HEV71/C7, were performed against OC/00219, OC/00260, and OC/00261, but neither serum neutralized the viruses (Table 4).

**Table 4:** Results of re-microneutralization tests with human enterovirus (HEV) monospecific antiserum, Osaka, Japan, 2000

Strain	Isolated cells	Predicted HEV Serotype^a^	HEV monospecific antiserum		
Anti-EV18	Anti- HEV71/BrCr	Anti- HEV71/C7
OC/0071	RD-18S	EV18^b^	+	ND	ND
OC/0073	RD-18S	EV18	+	ND	ND
OC/00219	Vero	HEV71	ND	--	--
OC/00260	Vero	HEV71	ND	--	--
OC/00261	Vero	HEV71	ND	--	--
OC/00272	RD-18S	EV18	+	ND	ND

^a^Serotypes were predicted from the phylogenetic analysis in Figure 1. ^b^ EV18 = echovirus 18; ND = Test not done.

### Phylogenetic Analysis of HEV71 Strains

**Figure 2:**
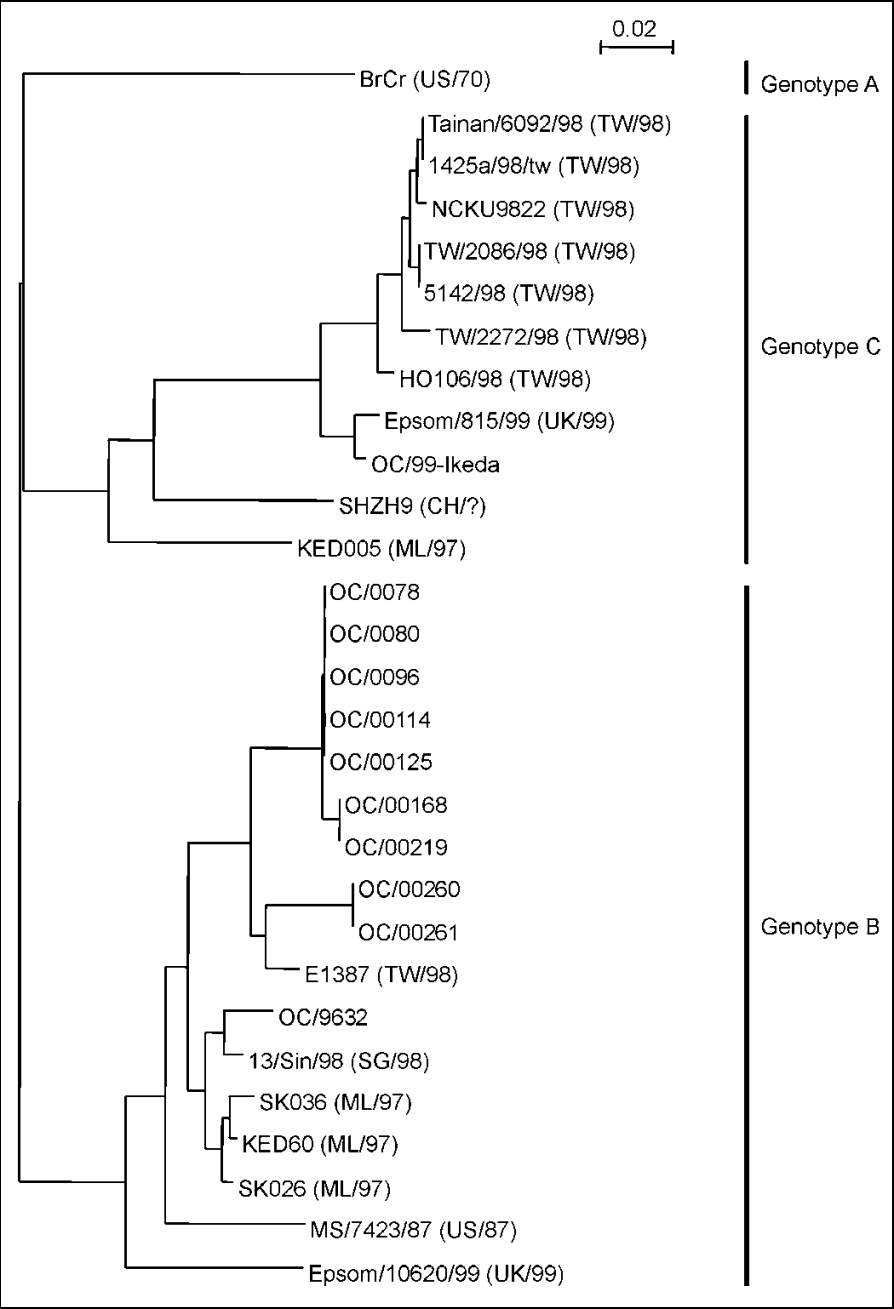
Phylogenetic analysis based on the human enterovirus 71 (HEV71) VP4 nucleotide sequences. The phylogenetic tree was constructed as described in the Figure 1 legend. The genotypes of the HEV71 cluster are denoted on the right. The VP4 sequences of 18 HEV71 strains available from GenBank are denoted by strain name, followed by the country and year isolated. Abbreviations for countries are as follows: US, United States; ML, Malaysia; CH, China; UK, United Kingdom; TW, Taiwan; and SG, Singapore. The accession numbers are as follows: BrCr; U22521, Tainan/6092/98; AF304459, 1425a/98/tw; AF176044, NCKU9822; AF136379, TW/2086/98; AF119796, 5142/98; AB037251, TW/2272/98; AF119795, HO106/98; AB037252, Epsom/815/99; AJ296213, SHZH9; AF302996, KED005; AB051334, E1387; AB051313, 13/Sin/98; AF251358, SK036; AB051333, KED60; AB051335, SK026; AB051332, MS/7423/87; U22522, Epsom/10620/99; and AJ296214.

To establish whether OC/00219, OC/00260, and OC/00261 belong to HEV71, we used another phylogenetic analysis based on the complete VP4 nucleotide sequences of various HEV71 strains (Figure 2). In this analysis, we examined eight HEV71 strains isolated and identified in our laboratory from 1996 to 2000 (Table 3). All these HEV71 strains except for OC/9632 were identified by microneutralization tests with anti HEV71/BrCr serum (data not shown). Of the HEV71 strains available from GenBank, two were isolated in the United States in 1970 and 1987, respectively (*25*), four in Malaysia in 1997 (*15*), one in Singapore in 1998 (*28*), eight in Taiwan in 1998 (*15*,*28*), two in the United Kingdom in 1999, and one in China (year unknown). The HEV71 strains were clustered in three distinct genotypes, designated A, B, and C. The genotype nomenclature of HEV71 strains for phylogenetic analyses based on the VP1 (*17*,*25*,*28*) and VP4 (*29*) nucleotide sequences has been reported, and the results (Figure 2) were consistent with previous findings. Among the HEV71 strains that were identified in our laboratory, only OC/99-Ikeda was classified in genotype C. Seven of eight strains identified in our laboratory by neutralization tests were classified in genotype B; five of these had the same VP4 nucleotide sequence. OC/00219, OC/00260, and OC/00261 were also classified in this genotype. This result demonstrated that strains OC/00219, OC/00260, and OC/00261 were HEV71 serotypes.

### Comparison of the Complete VP1 Nucleotide and Deduced Amino Acid Sequences of HEV71 Strains

Strains OC/00219, OC/00260, and OC/00261 were classified in HEV71 by the phylogenetic analysis, although these viruses were not neutralized by monospecific anti HEV71 sera. Because the VP1 protein contains a number of important neutralization sites (*1*,*30*), we determined the complete VP1 nucleotide sequences and compared the deduced amino acid sequences of OC/00219, OC/00260, and OC/00261. OC/00168 used as a prototype strain for HEV71 was also analyzed because this strain was neutralized by anti-HEV71/BrCr serum; moreover, its VP4 gene was the same as that of OC/00219 (Figure 2). The complete VP1 nucleotide sequences of these strains were 891 nt long, and the deduced amino acid sequences were 297 amino acids long. The differences of VP1 nucleotide sequences were 4 to 42 nt (0.4% to 4.7%), and the difference of deduced amino acid sequences was one amino acid (0.3%) among these viruses. The differences of VP1 nucleotide sequences between OC/00168 and OC/00219 were 4 nt (0.4%), and the deduced VP1 amino acid sequences of these strains were the same. The VP1 nucleotide and deduced amino acid sequences of OC/00260 and OC/00261 were identical (data not shown). The deduced VP1 amino acid sequences of these four strains were compared with that of BrCr (Table 5). There were 18 amino acid (6%) differences between BrCr and other strains. The different amino acid positions of strains OC/00168, OC/00219, OC/00260, and OC/00261 against BrCr were the same. Any mutated residues distinguishable BrCr and OC/00168 from OC/00219, OC/00260, and OC/00261 were not recognized in the VP1 amino acid sequences.

**Table 5:** Differences in deduced VP1 amino acid sequences (aa 1-297) of human enterovirus 71 strains BrCr, OC/00168, OC/00219, OC/00260, and OC/00261, Osaka, Japan, 2000

Amino acid position	Strain				
	BrCr^a^	OC/00168	OC/00219	OC/00260	OC/00261
18	Lys	Arg	Arg	Arg	Arg
22	Pro	Gln	Gln	Gln	Gln
30	Pro	Gln	Gln	Gln	Gln
31	Asp	Asn	Asn	Asn	Asn
43	Lys	Glu	Glu	Glu	Glu
58	Ala	Thr	Thr	Thr	Thr
98	Lys	Glu	Glu	Glu	Glu
145	Arg	Glu	Glu	Glu	Glu
164	Asp	Glu	Glu	Glu	Glu
167	Asp	Glu	Glu	Glu	Glu
172	Pro	Gln	Gln	Gln	Gln
183	Ser	Leu	Leu	Leu	Leu
184	Ser	Thr	Thr	Thr	Thr
244	Glu	Lys	Lys	Lys	Lys
246	Ser	Pro	Pro	Pro	Pro
249	Ile	Val	Val	Val	Val
275	Ser	Ala	Ala	Ala	Ala
282	Asp	Asn	Asn	Ser	Ser

^a^VP1 nucleotide sequence was obtained from GenBank (accession no. U22521) and translated into the deduced amino acid sequence.

## Discussion

The serotype identification of HEVs has been performed by microneutralization tests using standard HEV antiserum pools (*1*,*10*). Since >60 serotypes of HEV are known to infect humans (*1*,*19*), the HEV serotype is almost impossible to identify by using monospecific antiserum from the first microneutralization test. Furthermore, the neutralization test is labor-intensive and time-consuming, requiring several weeks. As an alternative, identification based on nucleotide sequences has been used successfully in several laboratories (*15*,*19*,*20*,*23*,*29*,*31*–*35*). To investigate the serotypes of the six untypeable HEV-like viruses that were not neutralized by the standard HEV antisera, we used phylogenetic analyses based on the complete VP4 nucleotide sequences of HEVs and were able to determine the serotype of each virus in the light of these results. OC/0071, OC/0073, and OC/00272 were thought to be EV18 strains by the phylogenetic analysis (Figure 1) and were neutralized by the monospecific anti-EV18 serum. These results indicate that the phylogenetic analysis based on the VP4 nucleotide sequence is consistent with the result of the microneutralization tests using the serotype-specific sera. OC/00219, OC/00260, and OC/00261 were thought to be HEV71 strains by the same analysis (Figure 1), but these viruses were not neutralized by the two monospecific anti-HEV71 sera. The phylogenetic analysis based on the HEV71 VP4 sequences confirmed that these viruses were HEV71 strains belonging to genotype B (Figure 2). We considered that OC/00219, OC/00260, and OC/00261 were all HEV71 strains not neutralized by anti- HEV71/BrCr and anti-HEV71/C7 sera, both available as standard monospecific anti HEV71 serum in Japan. These results also indicate that phylogenetic analysis with the VP4 sequences of HEVs can identify the serotypes in the same way as neutralization tests with HEV serotype-specific antisera. We are now preparing antiimmune sera against OC/00219, OC/00260, and OC/00261, respectively, to confirm antigenically that these are the prime strains of HEV71 neutralized by anti-HEV71/BrCr serum.

Oberste et al. have shown that HEV VP1 nucleotide sequences correlate with antigenically defined serotypes and have demonstrated the utility of VP1 sequences as a molecular surrogate for antigenic type (*19*,*35*). They have also shown that the VP1 sequences have a better correlation with HEV serotypes than the 5' NTR or the VP4-VP2 junction (*36*). The phylogenetic analysis based on the VP4 sequences we have described also correlates well with HEV serotypings by antiimmune sera. We used 21 HEV serotypes antigenically defined in our laboratory and another 8 strains available from GenBank as prototype strains in this analysis. We do not know whether 29 serotypes are sufficient for the phylogenetic analysis of HEV, as there are >60 serotypes. The good result of HEV phylogenetic classification based on the VP4 sequences might depend on the prototype numbers (29 of 64 serotypes) that we used. Ishiko et al., who performed HEV phylogenetic analyses based on VP4 sequences (*23*), used 45 HEV serotypes as prototype strains and obtained a phylogenetic tree similar to ours (Figure 1) except for a difference in the prototype strain numbers. Another phylogenetic analysis based on the VP4 sequences in this article was performed against the HEV71 strains (Figure 2). For this analysis, the HEV71 strains were clustered in three distinct genotypes, and the nomenclature was almost the same as for the HEV71 analyses based on the VP1 nucleotide sequences (*17**,**25*)*.* Recently, Chu et al. also reported the appropriateness of the phylogenetic analysis with the VP4 sequences for the molecular epidemiology of HEV71 outbreak in Taiwan in 1998 (*29*). These results suggest that the phylogenetic analysis based on the VP4 nucleotide sequences is also useful as a molecular surrogate for antigenic HEV serotyping. The analysis was more convenient based on the VP4 sequences than the VP1 sequences, since the complete VP4 sequence is 207 nt and the complete VP1 sequences are 834 to 951 nt (*35*), although the 3’ third of the VP1 sequence of 365 nt was used (*32*).

The VP4 nucleotide sequences of OC/99-Hanasaka and OC/00272 were identical, but the results of neutralization assays were different. OC/99-Hanasaka was easily neutralized by HEV pooled sera against EV18, but OC/00272 was not. The same results were observed for strains OC/00168 and OC/00219 HEV71, i.e., the results of their neutralization tests differed in spite of the VP4 sequence identity. These results indicate that the VP4 nucleotide sequences are highly conserved even though the neutralizable epitopes are antigenic variants. We compared the VP4 nucleotide and deduced amino acid differences of HEV71 strains, BrCr (*25*), E1387 (*15*), OC/9632, OC/99-Ikeda, OC/0078, OC/00219, and OC/00260. HEV71 genotypes indicated 1 to 37 nt (0.5% to 17.9%) differences. However, we found no amino acid differences (100% identity) (Table 6). Complete homology of the HEV71 VP4-deduced amino acid sequences has also been described (*20*,*29*), and Singh et al. demonstrated amino acid substitutions in the VP2 and VP3 regions, with the greatest variation in VP1 (*20*). These results indicate that VP4 is the most stable protein; accordingly, VP4 genes will be suitable for the molecular identification of HEV serotypes in the future.

**Table 6:** Number of nucleotide and deduced amino acid differences between the VP4 genes of human enterovirus 71 strains,^a^ Osaka, Japan, 2000

	BrCr	E1387	OC/9632	OC/0078	OC/00219	OC/00260	OC/99-Ikeda
BrCr		33	37	36	37	32	34
E1387	0		10	7	8	7	33
OC/9632	0	0		13	14	15	37
OC/0078	0	0	0		1	10	37
OC/00219	0	0	0	0		11	36
OC/00260	0	0	0	0	0		34
OC/99-Ikeda	0	0	0	0	0	0	
^a^Nucleotide numbers are given above the diagonal and amino acid numbers below it.							

VP4 is not exposed on the outer surface of the capsid, and no neutralizable epitopes appear to exist in VP4. On the other hand, VP1, VP2, and VP3 are outer capsid proteins and contain neutralizable epitopes (*37*,*38*). A number of important neutralization epitopes may exist on VP1 (*1*,*30*,*39*). To confirm the important neutralization sites on VP1, we compared the deduced VP1 amino acid sequences of HEV71 strains OC/00168, OC/00219, OC/00260, and OC/00261. OC/00168 was neutralized by anti-HEV71/BrCr serum, while OC/00219, OC/00260, and OC/00261 were not. Comparison of the deduced VP1 amino acid sequences showed that no mutated residues on the VP1 region corresponded to the result of the neutralization tests. This result indicates that either the important neutralization epiotopes for anti-HEV71/BrCr serum do not exist on the VP1 protein, or the epitopes are specifically masked in the cases of OC/00219, OC/00260, and OC/00261. Further analysis against the VP2 and VP3 regions of these strains should allow interpretation of these findings.
